# Heat-sensitive moxibustion self-administration in patients in the community with primary hypertension: A protocol for a multi-center, pragmatic, non-randomized trial

**DOI:** 10.1097/MD.0000000000022230

**Published:** 2020-09-18

**Authors:** Xu Zhou, Qingni Wu, Gaochuan Zhang, Yanping Wang, Shuqing Li, Baiyang Wang, Zhihua Chen, Weifeng Zhu, Fei Wang, Chun Gan

**Affiliations:** a Evidence-based Medicine Research Center, Jiangxi University of Traditional Chinese Medicine; b Honggutan Branch, Affiliated Hospital of Jiangxi University of Traditional Chinese Medicine; c The Second Affiliated Hospital of Jiangxi University of Traditional Chinese Medicine, Jiangxi, China.

**Keywords:** community health care, heat-sensitive moxibustion, pragmatic trial, primary hypertension, traditional Chinese medicine

## Abstract

**Background:**

Although the efficacy of antihypertensive drugs has been well established for primary hypertension, their effectiveness is always limited by side effects and poor compliance. Heat-sensitive moxibustion is an innovative acupoint stimulation therapy that is promising as a community health care intervention for hypertension.

**Aims:**

This study aims to evaluate the pragmatic effectiveness and safety of heat-sensitive moxibustion self-administration by patients in the community with primary hypertension.

**Methods:**

This study will adopt a multi-center, pragmatic, nonrandomized design. Six hundred patients with primary hypertension will be recruited from 4 communities. Each patient will choose to either receive heat-sensitive moxibustion self-administration + original antihypertensive drugs or maintain their original antihypertensive drugs without heat-sensitive moxibustion for 1 year.

**Expected outcomes:**

The primary outcome will be changes in systolic and diastolic blood pressures and the percentage changes in the doses of antihypertensive drugs. The secondary outcomes will be changes in quality of life assessed by a validated patient-reported outcome scale and the levels of fasting blood glucose, glycated hemoglobin, total cholesterol, triglycerides, low-density lipoprotein cholesterol, high-density lipoprotein cholesterol, urinary albumin, and serum creatinine. The proportion of patients with poor compliance with the heat-sensitive moxibustion regimen will also be evaluated as a secondary outcome. The safety of heat-sensitive moxibustion will be considered by analyzing the incidence of all and serious adverse events and their correlation with heat-sensitive moxibustion.

**Discussion:**

The findings of this study will provide pragmatic evidence for heat-sensitive moxibustion self-administration in patients in the community with primary hypertension and may also establish an ethical basis for further randomized controlled trials.

**Trial registration:**

The protocol of this trial was registered in ClinicalTrials.gov at May 11, 2020 (No. NCT04381520).

## Abstract 2

Supplemental Digital Content is available in the text

## 1 Introduction

Primary hypertension is a disease characterized by persistent high blood pressure without identifiable causes; it affects 31.1% of adults worldwide and is a chief cause of cardiovascular events and premature death.^[1]^ It is well established that blood pressure-lowering treatment can improve the prognosis of hypertension.^[2,3]^ However, the effectiveness of the mainstay of hypertension treatment, antihypertensive drugs, is always limited by side effects and poor compliance.^[4]^ Some studies even suggest that antihypertensive drugs are associated with increased risks of hip fracture and cancers (eg, skin cancer).^[5,6]^ Therefore, efforts are increasingly being made to research new management approaches for hypertension.

The efficacy of acupuncture and moxibustion for the treatment of hypertension has been suggested by a network meta-analysis of randomized controlled trials, in which moxibustion was shown to be more effective than acupuncture, although the evidence was of low quality.^[7]^ Moxibustion is an acupoint stimulation therapy that applies heat stimulation to specific acupoints by burning moxa.^[8]^ The antihypertensive mechanism of moxibustion is still uncertain. It is hypothesized that heat stimulation by moxibustion can result in the regulation of vasodilatation and cardiac rhythm, with mediators such as histamine, plasma aldosterone, and atrial natriuretic peptide; these effects, combined with the effects of the aroma and psychological relaxation experienced during moxibustion treatment, ultimately decrease blood pressure.^[9–11]^

Heat-sensitive moxibustion, an innovative acupoint stimulation therapy, has been developed based on traditional moxibustion in recent decades.^[12]^ The major innovation in heat-sensitive moxibustion therapy is the discovery of heat-sensitive acupoints.^[13]^ Researchers find that when persons suffer from diseases, heat-sensitive points appear on their body, and these points may differ from the classic acupoints in terms of location. When moxibustion is applied to heat-sensitive acupoints, patients experience unusual feelings (referred to as “heat-sensitive sensations”), including penetrating heat, expanding heat, heat transfer, distant heat, deep heat, and other feelings unrelated to heat (such as soreness, bloating, and numbness).^[12]^ It has been proven that heat-sensitive moxibustion is more efficacious than traditional moxibustion for multiple diseases, such as lumbar disc herniation and bronchial asthma.^[14,15]^

In principle, heat-sensitive moxibustion should be effective for hypertension. However, there is no high-quality evidence regarding the effects of heat-sensitive moxibustion on hypertension to date, and all current evidence regarding other acupoint stimulation therapies, including traditional moxibustion for hypertension, was generated in a hospital setting. For a chronic disease such as hypertension, compliance with treatment will be poor if patients are required to go to the hospital regularly to receive acupoint stimulation therapy. In contrast, patients can administer moxibustion treatment to themselves or with the help of their family at home after receiving brief training. Therefore, we assume that heat-sensitive moxibustion may be a feasible approach to the management of hypertension in a community setting. To verify this hypothesis, we will conduct a nonrandomized controlled study in multiple communities to evaluate the pragmatic effectiveness and safety of heat-sensitive moxibustion self-administration in patients with primary hypertension.

## 2 Methods and analysis

### 2.1 Study design

This is a multi-center, pragmatic, nonrandomized study that is registered in Clinicaltrials.gov (ID: NCT04381520) on May 11, 2020. This protocol (version 1.0) was developed according to the Standard Protocol Items: Recommendations for Interventional Trials (SPIRIT) checklist.^[16]^

### 2.2 Site and participant recruitment

**Figure 1:**
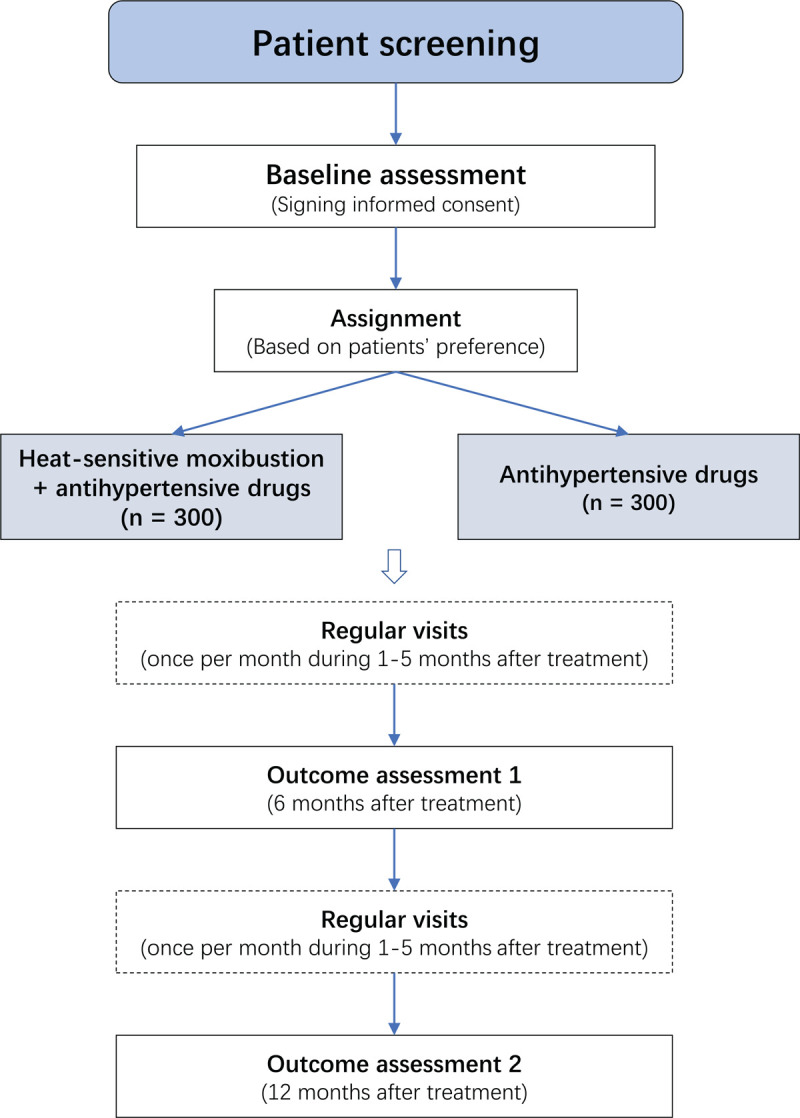
Flowchart of the study.

We will recruit patients with primary hypertension from 4 communities: the Nangang community, the Honggutan community, the Shengmi community in Nanchang city, and the Gaofu town community in Fu Zhou city. Recruitment will occur through community doctors’ oral communication with individuals, posters, and mobile advertising. Patients who express a preliminary willingness to participate in the study will be screened for eligibility and informed about the entire study process, benefits, and potential harms. Eligible patients will formally enter the study cohort after signing the informed consent form. The overall study design is shown in Figure 1.

### 2.3 Eligibility criteria

A patient will be eligible to participate in this study if he or she has been diagnosed with primary hypertension according to American College of Cardiology/American Heart Association criteria (ie, systolic blood pressure [SBP] ≥ 130 mm Hg or diastolic blood pressure [DBP] ≥ 90 mm Hg),^[17]^ is between 18 and 70 years old and is willing to sign the informed consent form. However, the following patients will be excluded:

(1) patients with hypertension secondary to pregnancy; brain, macrovascular, kidney or endocrine disease; or drug-induced factors;
(2) patients who are allergic to moxa, moxa smoke, or the moxibustion device;
(3) patients who are pregnant or lactating;
(4) patients who have experienced serious cardiovascular and cerebrovascular events (eg, myocardial infarction and stroke);
(5) patients with other severe diseases, such as liver and kidney dysfunction indicated by a level more than 2 times the upper limit of normal for total bilirubin, alanine aminotransferase, aspartate aminotransferase or serum creatinine; malignant tumors; and major mental disorders.

### 2.4 Training and assignment

There is a community health service center in each community. Before the study begins, we will invite senior experts to give 4 lectures (once per week) for residents in each community health service center to introduce the principal and anticipated efficacy of the use of heat-sensitive moxibustion for the management of hypertension. After fully comprehending the relevant knowledge, eligible patients will enter the heat-sensitive moxibustion group or the control group according to their preferences. Investigators and community doctors will not provide any advice regarding the group selection. For patients entering the heat-sensitive moxibustion group, community doctors will further provide one-on-one training, explaining how to perform heat-sensitive moxibustion, how to identify heat-sensitive acupoints, and how to avoid burns, among other safety precautions.

### 2.5 Interventions and co-interventions

**Figure 2:**
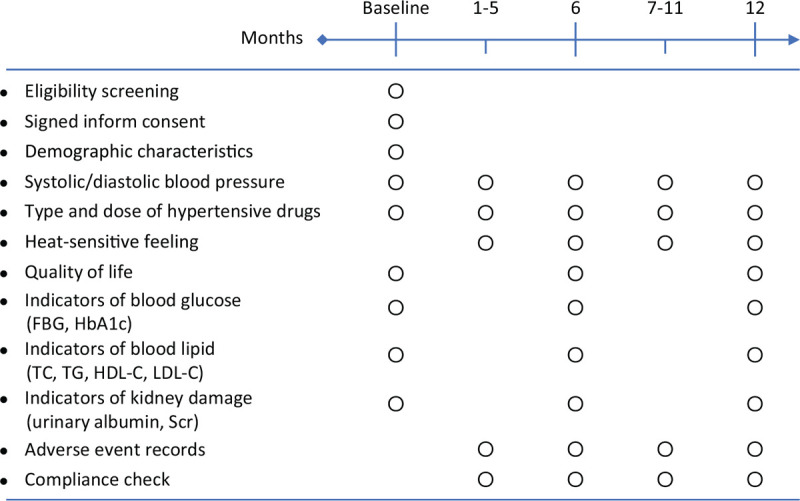
Schedule of study visits. FBG = fasting blood glucose, HbA1c = hemoglobin A1c, TC = total cholesterol, TG = triglycerides, HDL-C = high density lipoprotein cholesterol, LDL-C = low density lipoprotein cholesterol, Scr = serum creatinine.

Patients in the heat-sensitive moxibustion group will self-administer heat-sensitive moxibustion for one year while maintaining their original antihypertensive drugs. With the doctors’ help, the patients will first identify the heat-sensitive acupuncture points near CV8 (Shenque, the center of the navel), CV4 (Guanyuan, approximately 10 cm below the navel), and bilateral ST36 (Zusanli, approximately 7 cm below the lateral of the knee) and then perform moxibustion on the heat-sensitive acupoints. Self-sticking mini-moxa rolls will be used as the combustible material, which can be placed and burned in a moxibustion box (Fig. 2). A matching velvet bag can be used to wrap the moxibustion box to avoid burns. The suggested dose of moxibustion will be one moxa roll (approximately 30 minutes) per session, with 1 session per day and 7 sessions per week. Driven by a pragmatic design, we will allow the patients to adjust the dose of moxibustion to make it convenient, but they need to truthfully report the adjusted dose. Patients in the control group will only maintain their original antihypertensive drugs and will not perform heat-sensitive moxibustion. There will be no restriction on the type and dose of antihypertensive drugs, but other acupoint stimulation therapies, such as acupuncture, will be prohibited.

### 2.6 Outcome

#### 2.6.1 Primary outcomes

(1) Changes in SBP and DBP (mm Hg) measured with an electronic sphygmomanometer. The average value of 3 measurements will be used.
(2) Percentage changes in the dose of antihypertensive drugs. At each regular visit, the community doctors will determine whether the dose of antihypertensive drugs needs to be adjusted according to changes in blood pressure during the last month and will record the adjustment.

#### 2.6.2 Secondary outcomes

(1) Quality of life will be assessed by a validated patient-reported outcome scale,^[18]^ which includes 27 items and assesses the impacts of hypertension on the 3 dimensions of quality of life, namely, the physical dimension (18 items), psychological dimension (4 items), and family-social dimension (5 items). Each item is divided into 5 categories, with each category receiving 0 to 4 points in sequence. A higher score indicates a worse quality of life. The total score equals the sum of the score of each item, ranging from 0 to 108 points. The detailed content of the scale is provided in the Supplemental Content (http://links.lww.com/MD/E870). We will also analyze the scores of individual dimensions separately.
(2) Indicators related to diabetes mellitus, including changes in fasting blood glucose (mg/dl) and hemoglobin A1c (%), will be assessed.
(3) Indicators related to dyslipidemia, including changes in total cholesterol (mg/dl), triglycerides (mg/dl), low-density lipoprotein cholesterol (mg/dl), and high-density lipoprotein cholesterol (mg/dl) levels, will be assessed.
(4) Indicators related to hypertensive renal disease, including urinary albumin (g/l) and serum creatinine (μmol/l) levels, will be assessed.
(5) Patient compliance will be determined. Patient compliance will be deemed poor if a patient administers heat-sensitive moxibustion fewer than 8 times per month. The proportion of patients with poor compliance will be assessed.

### 2.7 Safety assessment

We will closely monitor the safety of heat-sensitive moxibustion throughout the study. Any adverse events (AEs) that are not related to the natural progression of hypertension will be recorded and reported to the Ethics Committee regularly. If a serious AE that leads to hospital admission, disability, a threat to life, or death occurs, we will report it to both the Ethics Committee and the Food and Drug Administration of Jiangxi Province within 24 hours.

We will also assess to what degree each AE is related to heat-sensitive moxibustion according to the following considerations^[19]^:

(1) whether there are time and position correlations between the AE and heat-sensitive moxibustion;
(2) whether the AE cannot be explained by other factors;
(3) whether the AE is alleviated or disappears when heat-sensitive moxibustion is stopped;
(4) whether the AE reappears when heat-sensitive moxibustion is administered again; and
(5) whether the AE can be explained by biological or pharmacological mechanisms.

### 2.8 Study visits

During the 1-year follow-up period, the investigators will visit the patients monthly at the community health service centers, which are generally within 1 km of the patients’ homes. At month 0 (baseline visit), we will collect demographic characteristics (name, phone number, sex, age, education level, height, and weight) and disease-related characteristics (SBP, DBP, course of hypertension, comorbidities, smoking, drinking, and type and dosage of antihypertensive drugs currently used). At months 6 and 12, we will measure blood pressure, changes in doses of antihypertensive drugs, quality of life, blood and urine indicators, and AEs. The other regular visits (ie, months 1–5 and 7–11) will involve the distribution of moxa rolls and moxibustion boxes to patients, the assessment of patient compliance and the adjustment of the doses of antihypertensive drugs. A detailed schedule of the study visits is shown in Figure 2.

### 2.9 Compliance enhancement

By default, we will provide patients in the heat-sensitive moxibustion group with 120 moxa rolls and 4 moxibustion boxes for free each month. If the moxibustion materials are insufficient because some are lost or for other reasons, patients can apply to receive more materials for free. When the patients return for a visit, they need to return the moxibustion box used last month to receive new moxibustion boxes. The investigators will determine whether the dose of moxibustion reported by the patients is consistent with the degree of blackening of the moxibustion boxes by the soot produced by burning the moxa. Patients in both groups will receive free health consultations and physical examinations at each visit and a reward of 100 and 200 Chinese yuan when the 6- and 12-month visits are completed, respectively. Of course, patients can voluntarily withdraw whenever for whatever reason during the study, and withdrawal will not affect any subsequent treatment.

### 2.10 Data management and monitoring

All investigators will receive training in standardized operating procedures before the study starts. Data will be collected by community doctors using pilot-tested case report forms. Each patient's informed consent form and case report forms will be bound into 1 volume for management. The distribution of the moxa rolls and moxibustion boxes will also be strictly recorded. The software used to manage the electronic database will be Microsoft Access version 2016 (Microsoft Corporation, US), and the data will be doubly input and cross-checked by 2 independent investigators. All data in the database will be anonymized. When performing data analysis, the grouping will be concealed from the analysts.

An independent data monitoring committee will be established to monitor the quality of the data collection (eg, whether there are any incorrectly reported or missing data) and to ensure that any amendments or incomplete data are addressed in a timely manner. The committee will also have the right to plan an interim analysis (eg, when all patients have completed 6 months of follow-up) and to decide whether to discontinue the study based on the efficacy and safety results of the interim analysis.

### 2.11 Sample size calculation

We used the expected reduction in SBP as a reference and a sample size formula for continuous outcomes in nonrandomized controlled studies to estimate the minimum required sample size. Based on the results of our pilot observations and experts’ advice, we expect to measure a 5 mm Hg change in SBP with a standard deviation of 20 mm Hg. Setting the probabilities of type I error and type I error of 0.05 and 0.20, respectively, and considering a 20% attrition rate, the minimum sample size is calculated to be 140 per group. As we have sufficient funding, we increase the expected sample size to 300 cases per group to allow adjustment for more confounders.

### 2.12 Statistical analysis plan

The analyses of the primary outcomes will be performed in the intention-to-treat population where either patients in the control group who performed heat-sensitive moxibustion or patients in the heat-sensitive moxibustion group who stopped moxibustion during follow-up will still be kept in their original group. Meanwhile, we will also perform sensitivity analyses using the perprotocol population to test the robustness of the main analysis. All analyses of the secondary outcomes will be performed in the per-protocol population. We will decide whether to impute missing data and which method should be used to impute missing data based on the actual distribution of the missing data.

For categorical or ordinal variables, we will calculate frequencies and percentages as the descriptive statistics and compare the between-group differences using the chi-square test or the linear-by-linear association test. We will present the means with standard deviations/errors and medians with interquartile ranges for continuous variables with normal and skewed distributions, respectively; the independent *t* test or the Mann-Whitney *U* test will be used to compare 2 groups. The effects of heat-sensitive moxibustion on the outcomes will be inferred using analysis of covariance or a logistic regression model adjusted for age, course of hypertension, baseline SBP, comorbidities, education level, smoking status, drinking status, and types of antihypertensive drugs recoded as dummies. The effects will be measured as the mean difference or odds ratio with a 95% confidence interval, and *P*-values will also be calculated. In addition, we will test 2 subgroup hypotheses:

(1) the dose of moxibustion: ≥3 sessions/wk vs <3 sessions/wk;
(2) whether the heat-sensitive sensation appears: yes vs no.

The significance level indicating a difference between groups and subgroups will be α = 0.05. We will use R version 4.0.2 (The R Foundation for Statistical Computing, Austria) to run all statistical analyses.

### 2.13 Dissemination plan

The trial results will be disseminated through webpages and publications in peer-reviewed journals.

### 2.14 Ethics approval

This trial has been approved by the ethics committee of the Second Affiliated Hospital of Jiangxi University of Traditional Chinese Medicine (No. 2019-016). The trial will be conducted in compliance with the Declaration of Helsinki and the principles of Good Clinical Practices. Written informed consent will be obtained from all participants.

## 3 Discussion

Although acupuncture has long been used to treat hypertension, and there is no lack of evidence supporting its efficacy, it is invasive and must be performed by qualified doctors.^[20]^ Therefore, acupuncture cannot be self-administered by patients or used as a long-term therapy. Heat-sensitive sensitive moxibustion is a noninvasive therapy, and patients can easily learn how to administer it and reach an expected level of efficacy after training. Self-administered moxibustion is also quite inexpensive, costing only approximately 30 Chinese yuan (4 dollars) per month. If our study verifies that heat-sensitive moxibustion does have a significant efficacy for the treatment of hypertension, it may be able to partially replace antihypertensive drugs, reduce medical costs, and serve as a long-term therapy in the community setting. Although a randomized controlled trial is the gold standard for establishing the efficacy of any therapy, it may be ethically problematic without preexisting efficacy and safety evidence. Therefore, this nonrandomized pragmatic study also aims to provide an ethical basis for further randomized controlled trials.

In 2017, the government of Xiashan District in Weifang city in Shandong Province tried for the first time to popularize heat-sensitive moxibustion as a daily health care method for all residents of the town. The measures taken to popularize the knowledge of heat-sensitive moxibustion included building a community center dedicated to providing free sessions of heat-sensitive moxibustion, organizing expert lectures, training patients on-site, and providing free moxibustion materials. After 2 years of efforts, the popularization work achieved satisfactory results – heat-sensitive moxibustion was practiced by 67% of the total population of 62,000 people. In addition, the per capita medical expenditure decreased by 49.8% and the number of annual outpatient consultations decreased by more than 16.7% compared with the values 2 years before.^[21]^ Unfortunately, no rigorous evidence was obtained regarding the effects of heat-sensitive moxibustion on hypertension with the exception of scattered case reports published during the popularization campaign. Learning from the successful experience in Shandong Province, the government of Jiangxi Province started to promote a public health project called “Everyone Uses Heat-sensitive Moxibustion, Everyone Enjoys Health” in several cities in 2019. Currently, the project is progressing smoothly. It could provide a favorable climate for the recruitment of participants and the successful completion of this study.

This study has potential limitations. The reduction of internal validity caused by the nonrandomized design is the main limitation. Although we have expanded the expected sample size as much as possible and will adjust for multiple confounders in the statistical analysis, the bias caused by unknown confounders cannot be completely avoided. However, the extrapolation of the results of this study, as a major advantage of a pragmatic study, will be enhanced. Another limitation is that it may be difficult for patients to remain compliant with the heat-sensitive moxibustion protocol for 1 year, and poor compliance will introduce bias. However, an important purpose of this study is to evaluate the feasibility of heat-sensitive moxibustion self-administration as a long-term intervention in patients in the community with hypertension. If compliance is poor, it will show that this is not a feasible strategy.

In summary, with the increasing incidence of hypertension, it is imperative to emphasize community-based management. Heat-sensitive moxibustion self-administration has potential advantages in terms of efficacy and compliance. The findings of this study will provide the first pragmatic evidence of the effect of heat-sensitive moxibustion self-administration by patients in the community on primary hypertension and may impact the government's public health decisions.

## Author contributions

**Conceptualization:** Fei Wang, Chun Gan.

**Investigation:** Qingni Wu, Gaochuan Zhang, Yanping Wang, Shuqing Li.

**Methodology:** Xu Zhou, Baiyang Wang, Zhihua Chen.

**Writing – original draft:** Xu Zhou.

**Writing – review & editing:** Weifeng Zhu, Fei Wang, Chun Gan.

## Abbreviations

Abbreviations: AE = adverse event, DBP = diastolic blood pressure, SBP = systolic blood pressure.
